# Editorial: Pathogen-host interaction in the development of viral hepatitis

**DOI:** 10.3389/fcimb.2023.1333470

**Published:** 2023-11-21

**Authors:** Shuxiang Li, Jiarui Li, Yunyang Xu, Ze Xiang, Jian Wu

**Affiliations:** ^1^ Department of Clinical Laboratory, The Affiliated Suzhou Hospital of Nanjing Medical University, Suzhou Municipal Hospital, Gusu School, Nanjing Medical University, Suzhou, China; ^2^ Zhejiang University, School of Medicine, Hangzhou, China

**Keywords:** viral hepatitis, progress, challenges, control, management

Inflammation of the liver parenchyma caused by viral infection is called viral hepatitis. Hepatotropic viruses, including hepatitis A virus (HAV), hepatitis B virus (HBV), hepatitis C virus (HCV), hepatitis D virus (HDV) and hepatitis E virus (HEV), constitute the vast majority of viral hepatitis infections (Xiang et al., 2022b). Viral hepatitis is one of the diseases with the highest global burden. Millions of people worldwide are affected by viral hepatitis every year. The clinical spectrum of the disease ranges from asymptomatic to acute hepatitis, acute liver failure, chronic liver disease, and even hepatocellular carcinoma (HCC). Based on the great threat posed by viral hepatitis and the prospect of research, the World Health Organization (WHO) put forward an initiative to reduce viral hepatitis infections by 90% by 2030.

HAV was first identified in 1973 and is transmitted by the fecal-oral route. Acute hepatitis A is usually benign and resolves spontaneously in most patients, thus, limited data make specific antiviral agents against HAV infection currently unavailable (Shin and Jeong, 2018). Therefore, it is particularly important to develop a cell line against HAV infection. Win et al. successfully supported HAV replication using human liver PXB cells derived from a human-derived chimeric mouse model and found that Japanese miso extract made from rice koji enhanced the expression of glucose-regulated protein 78 and exerted anti-HAV effects (Win et al., 2018). HAV vaccine is the more widely used method in the prevention and treatment of the virus, and different types of HAV vaccines have been evaluated. A phase 3 trial testing the immunogenicity and safety of coadministration of a quadrivalent live attenuated dengue vaccine and an inactivated HAV vaccine showed good tolerance and significant immune responses to coadministration of both types of vaccines (Tricou et al., 2023).

HBV infection is the leading cause of chronic liver disease worldwide, with an estimated 296 million cases of chronic hepatitis B globally (Akinyemiju et al., 2017). Although a preventive vaccine and antiviral therapy are available to inhibit HBV replication, there is no cure. The need to develop curative HBV therapies calls for new biomarkers for monitoring viral and host responses. The International Coalition to Eliminate of HBV pointed out that emerging biomarkers such as HBV RNA and hepatitis B core-related antigen have the potential to predict the development of hepatitis B to chronic liver disease and evaluate the response to antiviral treatment (Kramvis et al., 2022). In addition, the current animal model of HBV infection is still not optimal. Revill et al. indicated that further development of a dual humanized mouse model of the liver and immune system would allow the study of immune-mediated clearance (Revill et al., 2019). Kah et al. used HBV-infected human hepatic chimeric uPA/SCID/ILγR2 mice and found that engineered T cells briefly expressing HBV-TCR promptly mediated virus-specific immune responses and reduced the intrahepatic viral load in mice (Kah et al., 2017).

HCV was first reported in 1989. The WHO estimates that in 2019, 290 000 people died from cirrhosis and HCC due to chronic HCV infection. The approval of direct-acting antivirals in 2014 has revolutionized the treatment of hepatitis C with their sustained virologic response and greatly improved the cure rate of hepatitis C (Mizokami et al., 2015). However, there is currently no effective HCV vaccine (Ray, 2011). A randomized trial of a vaccine to prevent chronic HCV infection showed that the vaccine produced HCV-specific T-cell responses that reduced peak HCV RNA levels but did not prevent chronic HCV infection (Page et al., 2021). At the same time, it is still necessary to educate people, especially in third world countries, about the transmission of HCV infection if worldwide elimination of HCV is to be achieved and the latest anti-HCV drugs should be provided in a timely manner (Taherkhani and Farshadpour, 2017).

HDV is a highly pathogenic virus that causes acute, fulminant, and chronic hepatitis, causing cirrhosis in more than 70% of cases. As a defective virus, HDV is unable to synthesize its envelope proteins independently and therefore requires HBV surface antigens in host cells to complete its replication (Farci and Anna Niro, 2018). The exploration of suitable drugs for HDV infection is still a research hotspot. A recent phase 3 trial tested the efficacy of bulevirtide (BLV) in controlling the progression of chronic qualitative hepatitis-related liver disease, and BLV treatment significantly reduced HDV RNA levels and improved alanine aminotransferase levels (Wedemeyer et al., 2023). Similarly, the safety and efficacy of BLV monotherapy in HDV-related compensated cirrhosis and clinically significant portal hypertension have been demonstrated (Degasperi et al., 2022). Both lonafarnib monotherapy (Ullrich et al., 2013) and triple therapy with lonafarnib, ritonavir, and peginterferon lambda-1a (Koh et al., 2020) have shown antiviral efficacy in patients with chronic HDV infection.

HEV is the most important causative agent of acute viral hepatitis. Infection in pregnant women can cause severe hepatitis, acute liver failure, and a mortality rate as high as 20%-25% (Xiang et al., 2022a). In addition, HEV infection can cause many extrahepatic manifestations (Wu et al., 2021). Currently, there are no approved effective available treatment regimens for HEV infection. There are still many challenges in the diagnosis and treatment of hepatitis E, such as screening and management, prevention and treatment of extrahepatic manifestations, vaccine evaluation, and establishment of animal models (Raji et al., 2022). There is a need for new therapeutic options for HEV infection in immunocompromised patients, and organic combinations of drugs such as ribavirin, sofosbuvir, and 2’-C-methylguanosine have the potential to be used (Nishiyama et al., 2019). HEV 239 is currently the only officially approved vaccine against HEV in the world. Evaluation of the immunogenicity and safety of a two-dose HEV 239 vaccine regimen in a population with a high burden of HEV1 infection revealed that the vaccine elicited a broad and possibly functional immune response against HEV (Øverbø et al., 2023).

In conclusion, the five strains of viral hepatitis differ significantly in transmission routes, geographical distribution, degree of development, and prevention methods. In recent years, through public education, screening management, vaccination and other interventions, viral hepatitis has been controlled to a certain extent, but the related management and control programs of viral hepatitis still need to be further explored, and there is a long way to go to achieve the goal of hepatitis-free world.

## Author contributions

SL: Writing – original draft. JL: Writing – original draft. YX: Writing – original draft. ZX: Writing – review & editing. JW: Writing – review & editing.
